# Supplementary material for: TGFα controls checkpoints in CNS resident and infiltrating immune cells to promote resolution of inflammation
Source: Nat Commun. 2025 Jun 19;16:5344. doi: 10.1038/s41467-025-60363-7 (PMC12179293; doi:10.1038/s41467-025-60363-7)
Supplement: Supplementary file 1 — Supplementary Information [file 41467_2025_60363_MOESM1_ESM.pdf]

SUPPLEMENTARY INFORMATION

TGFα controls checkpoints in CNS resident and infiltrating immune cells to promote resolution of inflammation

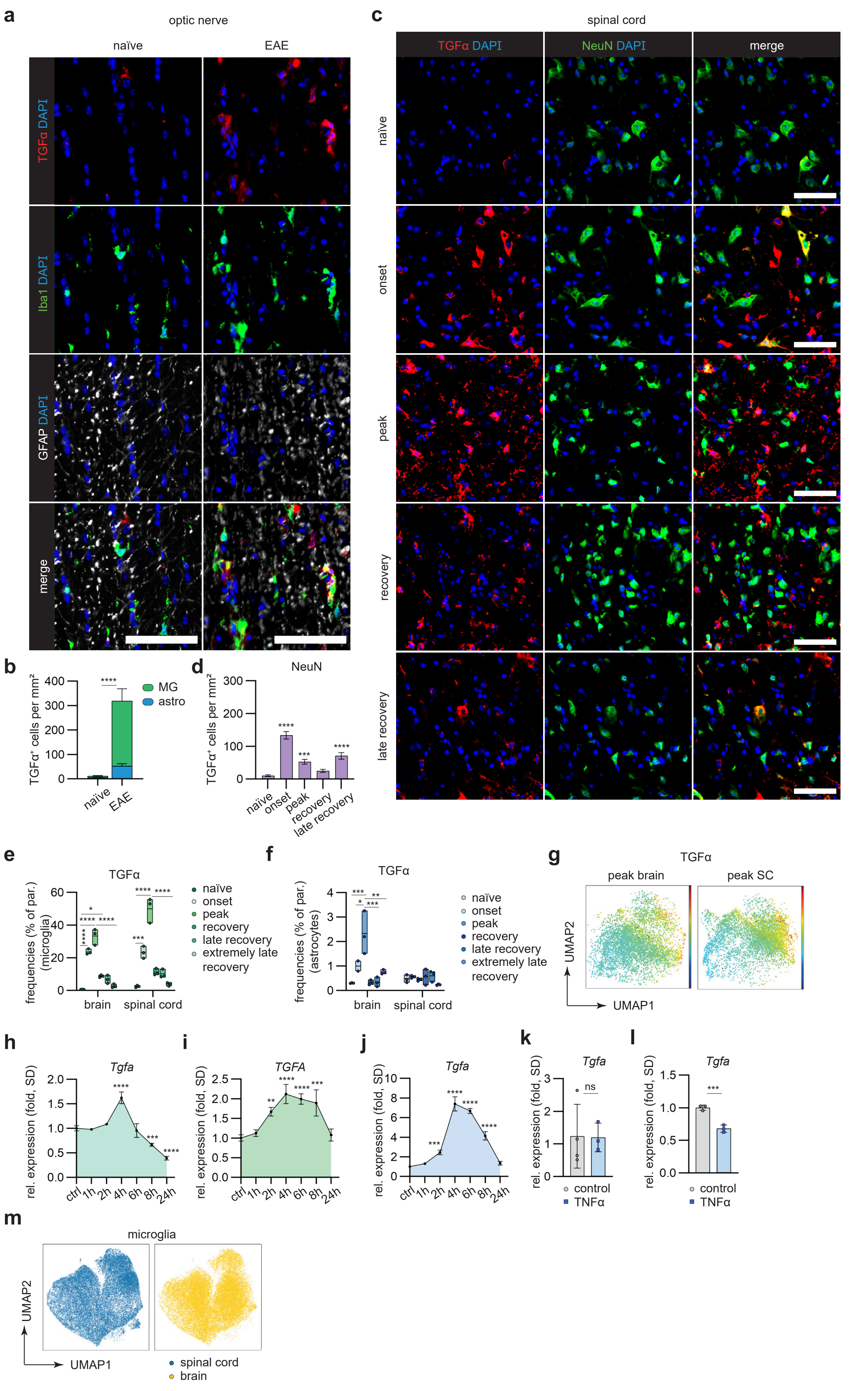

Supplementary Figure 1

Supplementary Figure 1. Spatiotemporal regulation of microglia-derived TGFα during acute CNS inflammation. **a**, Immunostaining and quantification (**b**) of TGFα+ Iba1+ and TGFα+ GFAP+ cells and DAPI for nuclear staining in the optic nerves of naïve and EAE mice. n= 24 per group. Scale bar 50 µm. **c**, Immunostaining and quantification (**d**) of TGFα+ NeuN+ cells and DAPI for nuclear staining in spinal cords of EAE (onset, peak, recovery, late recovery; n=3/5 per timepoint) and naïve mice (n=5). Scale bar 50 µm. **e,** Relative expression (% of parent) of TGFα+ microglia and astrocytes **(f**) in spinal cords and brains of EAE during onset, peak, recovery, late recovery, extremely late recovery and naïve mice (n=3 per timepoint) quantified by intracellular flow cytometry. Line at mean. **g**, UMAP plot of TGFα expression in subsampled microglia in the brain (left) and spinal cord (SC; right) at peak of EAE analysed by high-dimensional flow cytometry. **h**, RT-qPCR analysis of *Tgfa* expression in primary mouse microglia and human microglia (HMC3) (**i**) stimulated with LPS over timecourse (1h, 2h, 4h, 6h, 8h, 24h). n = 3 per group. **j**, RT-qPCR analysis of *Tgfa* expression in primary mouse astrocytes stimulated with TNFα/ IL-1β or vehicle over timecourse (1h, 2h, 4h, 6h, 8h, 24h). n = 4/6 per group. **k**, RT-qPCR analysis of *Tgfa* expression in neuronal cells (N2a) stimulated with TNFα. n = 3/4 per group. **l**, RT-qPCR analysis of *Tgfa* expression in primary mouse oligodendrocytes stimulated with TNFα. n = 3 per group. **m**, UMAP plot of subsampled microglia in the spinal cord (blue) and brain (yellow) during the course of EAE (n=5-6 per timepoint) analysed by high-dimensional flow cytometry. Data shown as mean ± SD. Data shown as mean ± SEM in (**b, d**). Two-way ANOVA with Sidak's multiple comparisons test in (**b**). Two-way ANOVA with Tukey's multiple comparisons test in (**e, f**). One-way ANOVA with Dunnett's multiple comparisons test in (**h**, **I, j**). Unpaired t-test in (**k, l**). $^{*}P < 0.05$; $^{**}P < 0.01$; $^{***}P < 0.001$; $^{****}P < 0.0001$.

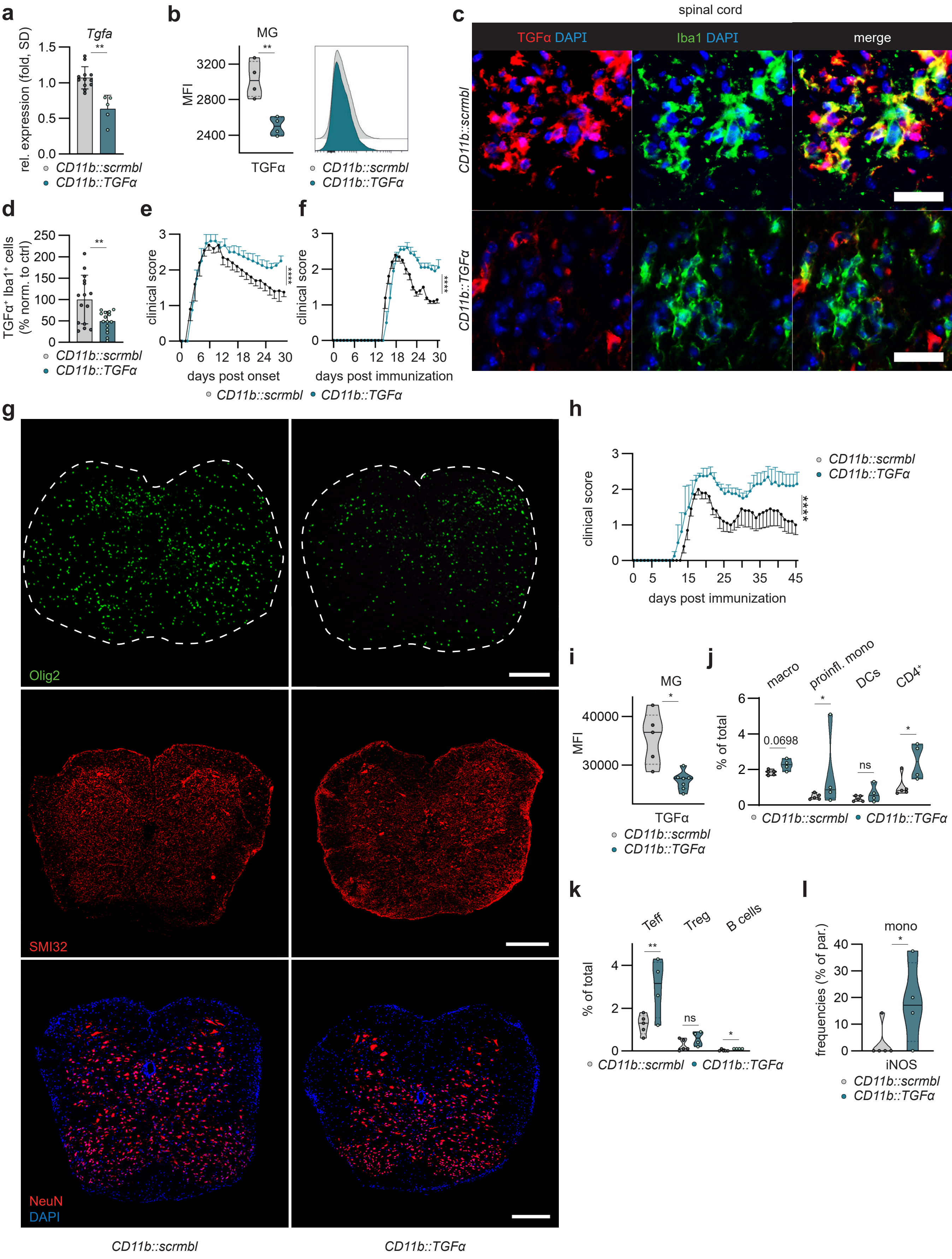

Supplementary Figure 2

Supplementary Figure 2. Microglial knock-out of TGFα during acute CNS inflammation. a, RT-qPCR analysis of *Tgfa* expression in sorted mouse microglia from the CNS of *CD11b::scrmbl* (n=11) and *CD11b::TGFα* (n=5) mice. **b**, Quantification (left) and histogram (right) of mean fluorescence intensity (MFI) of microglial (MG) TGFα expression in the CNS of *CD11b::scrmbl* and *CD11b::TGFα* mice analysed at day 30 by intracellular flow cytometry. n = 4 per group. **c**, Immunostaining and quantification (**d**) of TGFα+ Iba1+ cells and DAPI for nuclear staining in the spinal cord of *CD11b::scrmbl* (n=14) and *CD11b::TGFα* (n=15) mice. Scale bar 30 µm. **e**, EAE development in mice transduced with *CD11b::scrmbl* (n=5) or *CD11b::TGFα* (n=5) plotted from onset of disease. **f**, Delivery of lentiviruses via intracerebroventricular (i.c.v.) injection at peak of EAE and disease development in mice transduced with *CD11b::scrmbl* or *CD11b::TGFα* (n=5). **g**, Representative overview images of immunostaining of Olig2+ oligodendrocytes (top), SMI32 (middle) and NeuN+ neurons (bottom, DAPI as nuclear staining) in lumbar spinal cord of *CD11b::scrmbl* (n=5) and *CD11b::TGFα* (n=4) mice. **h,** EAE development until day 45 in mice transduced with *CD11b::scrmbl* (n=5) or *CD11b::TGFα* (n=4). **i,** Quantification of mean fluorescence intensity (MFI) of microglial (MG) TGFα expression in the CNS of *CD11b::scrmbl* and *CD11b::TGFα* mice analysed at day 45 by intracellular flow cytometry. n = 4 per group. **j,** Abundance of monocytes (mono), neutrophils (neutro), pro-inflammatory monocytes (mono), dendritic cells (DCs) and CD4+ T cells, and effector T cells (T eff), regulatory T cells (T reg), and B cells (**k**) in the CNS of *CD11b::scrmbl* (n=5) and *CD11b::TGFα* (n=4) mice (day 45). **l,** iNOS production by monocytes (mono) in the CNS of *CD11b::scrmbl* (n=5) and *CD11b::TGFα* (n=4) mice analysed by intracellular flow cytometry (day 45). Data shown as mean ± SD. Data shown as mean ± SEM in (**e, f, h**). Non-parametric Wilcoxin matched-pairs signed-rank test in (**e, f, h**). Unpaired t-test in (**a, b, d, i, j, k, l**). *$P < 0.05$; **$P < 0.01$; ***$P < 0.001$; ****$P < 0.0001$.

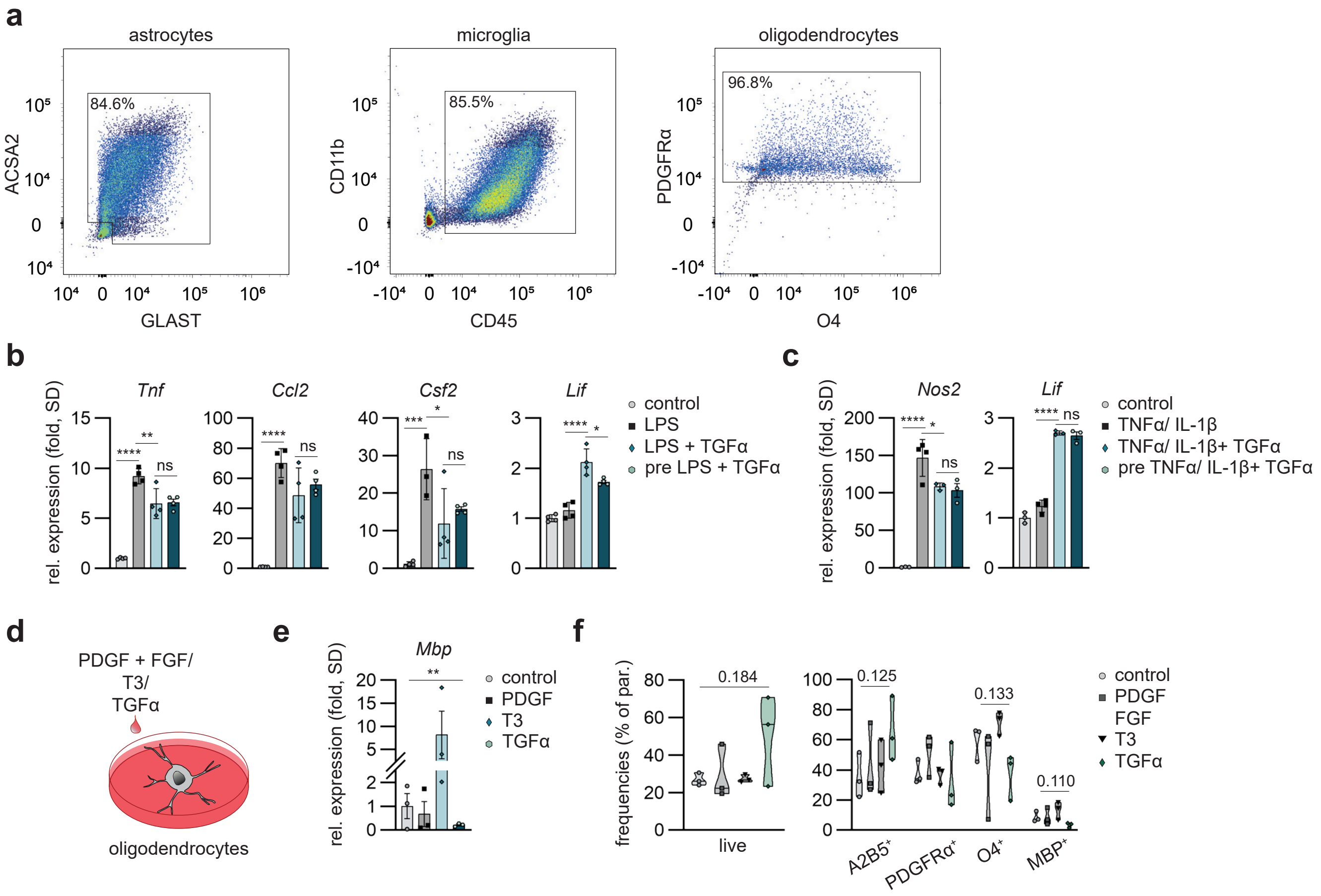

Supplementary Figure 3

Supplementary Figure 3. TGFα promotes protective effects under inflammatory and demyelinating conditions. **a,** Purity of primary glial cells after shake off (astrocytes, left), sorted microglia (middle) and oligodendrocytes (right). **b,** RT-qPCR analysis of *Tnf*, *Ccl2, Csf2* and *Lif* expression in primary mouse microglia stimulated with vehicle, LPS ± TGFα or pre-stimulation with LPS + LPS/ TGFα. n = 4 per group. **c**, RT-qPCR analysis of *Nos2* and *Lif* expression in primary mouse astrocytes stimulated with vehicle, TNFα/ IL-1β ± TGFα or pre-stimulation with TNFα/ IL-1β + TNFα/ IL-1β / TGFα. n = 4 per group. **d**, Schematic, RT-qPCR analysis of *Mbp* expression (**e**) and flow cytometric analysis (**f**) of primary mouse oligodendrocytes during differentiation ± TGFα, Triiodothyronine (T3), PDGF/FGF. n = 3 per group. **f**, Live (left) and flow cytometric quantification (right) of A2B5+, PDGFRα+, MBP+, and O4+ cells (% of singlets) during differentiation ± TGFα, Triiodothyronine (T3), PDGF/FGF. n = 3 per group. Data shown as mean ± SD. Data shown as mean ± SEM in (**e**). One-way ANOVA with Tukey's multiple comparisons test in (**b**, **c**, **e**, **f**). $^{*}P < 0.05$; $^{**}P < 0.01$; $^{***}P < 0.001$; $^{****}P < 0.0001$.

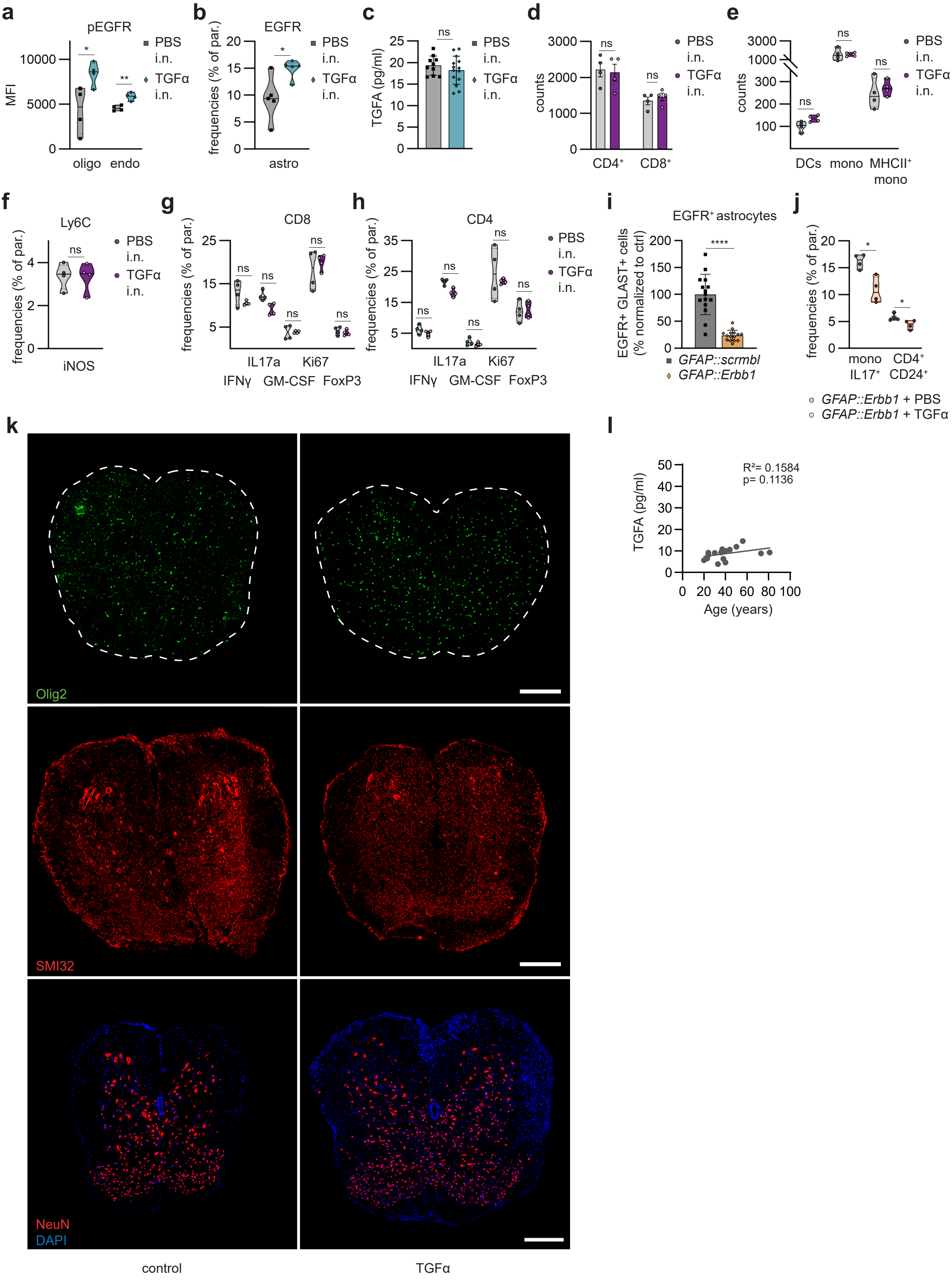

Supplementary Figure 4

Supplementary Figure 4. TGFα as treatment target for lesion resolution in autoimmune CNS inflammation. a, Quantification of mean fluorescence intensity of pEGFR expression in oligodendrocytes (oligo) and endothelial cells (endo) of the CNS from TGFα or vehicle treated EAE mice (intranasal) analysed by intracellular flow cytometry. n = 4 per group. **b,** CNS cells from TGFα or vehicle treated EAE mice (intranasal) analysed by high-dimensional flow cytometry showing relative expression levels (% of parent) of EGFR in astrocytes. n = 5 per group. **c**, Enzyme-linked Immunosorbent Assay (ELISA) of whole brain samples from TGFα or vehicle treated EAE mice (intranasal). **d-e,** Abundance of CD4+, CD8+ T cells (**d**) and dendritic cells (DC), monocytes (mono) and pro-inflammatory monocytes (MHCII+) (**e**) in spleen from TGFα or vehicle treated EAE mice (intranasal) analysed by high-dimensional flow cytometry. n = 4 per group. **f,** Relative expression (% of parent) of iNOS in Ly6C+ splenic monocytes from TGFα or vehicle treated EAE mice (intranasal) analysed by intracellular flow cytometry. n = 4 per group. **g**, Splenic cells from TGFα or vehicle treated EAE mice (intranasal) analysed by intracellular flow cytometry showing expression levels of proliferation (Ki67) marker, transcription factor (FoxP3) and cytokine production (IFNγ, IL17a, GM-CSF) in CD8+ and CD4+ (**h**) T cells. n = 4 per group. **i**, Quantification of EGFR+ GLAST+ cells in the lumbar spinal cord of *GFAP::scrmbl* (n=15) and *GFAP::Erbb1* (n=15) mice analysed by immunostaining. **j**, CNS cells from TGFα or vehicle treated EAE mice (intranasal; start day 7) transduced with *GFAP::Erbb1* (n=4) analysed by intracellular flow cytometry showing IL17+ production in monocytes and CD24+ production in CD4+ T cells. **k**, Representative overview images of immunostaining of Olig2+ oligodendrocytes (top), SMI32 (middle) and NeuN+ neurons (bottom, DAPI as nuclear staining) in lumbar spinal cord of symptomatic intranasal treatment with TGFα or vehicle. **l,** Solid line shows linear regression of correlation between TGFA concentration (pg/ml) measured by Enzyme-linked Immunosorbent Assay (ELISA) and age in control samples (n=17). Values are means of technical duplicate measurements. Data shown as mean ± SD. Data shown as mean ± SEM in (**d**). Unpaired t-test in (**a, b, c, i**). Two-way ANOVA with Sidak's multiple comparisons test in (**d**, **e**, **f**, **g**, **h, j**). *$P < 0.05$; **$P < 0.01$; ***$P < 0.001$; ****$P < 0.0001$

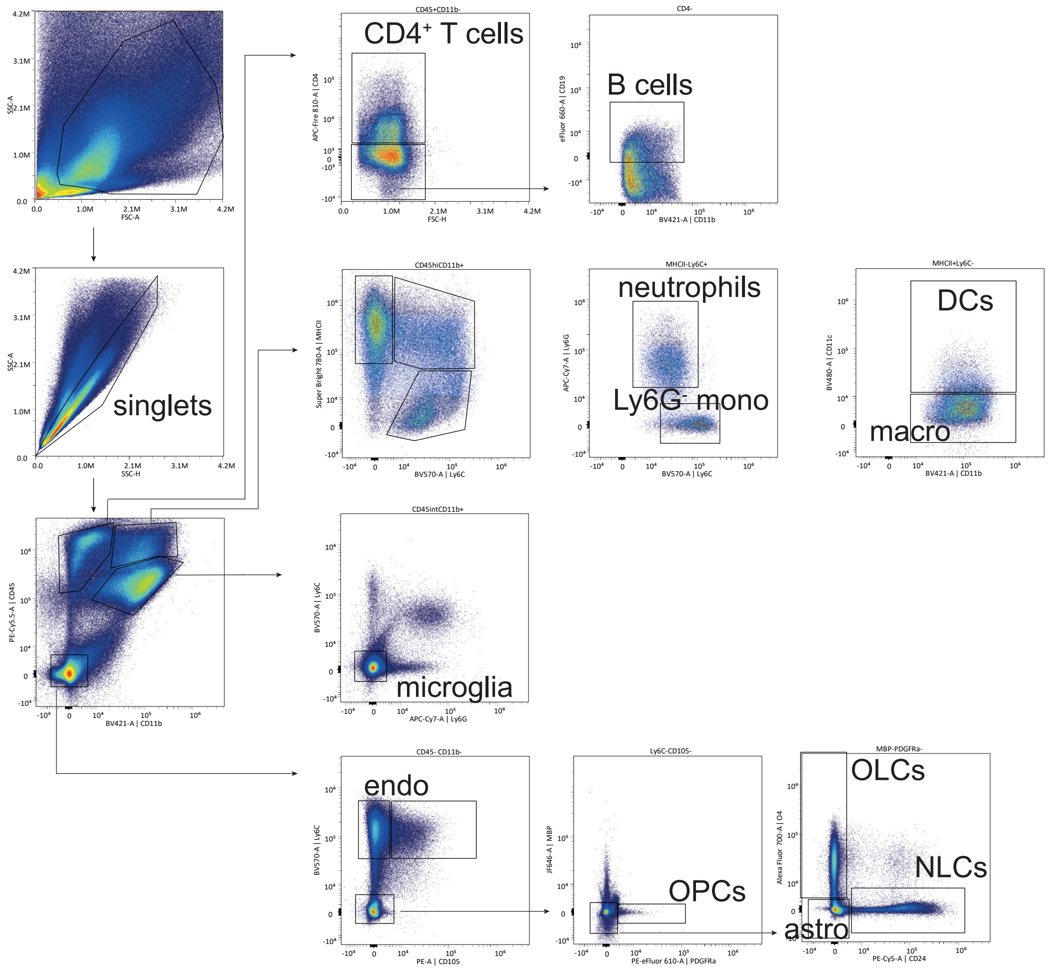

Supplementary Figure 5

Supplementary Figure 5. Gating strategy: Flow cytometry of CNS surface marker.

a

Patient #	Diagnosis	disease duration (in weeks)	EDSS	Immunmodulatory Therapy
1	Control	NA	NA	NA
2	Control	NA	NA	NA
3	Control	NA	NA	NA
4	Control	NA	NA	NA
5	Control	NA	NA	NA
6	Control	NA	NA	NA
7	Control	NA	NA	NA
8	Control	NA	NA	NA
9	Control	NA	NA	NA
10	Control	NA	NA	NA
11	Control	NA	NA	NA
12	Control	NA	NA	NA
13	Control	NA	NA	NA
14	Control	NA	NA	NA
15	Control	NA	NA	NA
16	Control	NA	NA	NA
17	Control	NA	NA	v
18	RRMS relapse	0	1	none
19	RRMS relapse	10	6.5	none
20	RRMS relapse	3	0	none
21	RRMS relapse	0	2	none
22	RRMS relapse	0	2	none
23	RRMS relapse	2	2	none
24	RRMS relapse	9	3	none
25	RRMS relapse	0	2	none
26	RRMS relapse	2	2.5	none
27	RRMS relapse	0	0	none

b

Patient #	Diagnosis	disease duration (in weeks)	EDSS	Immunmodulatory Therapy
1	RRMS	8	0	none
2	RRMS	10	0	none
3	RRMS	0	0	none
4	RRMS	12	0	none
5	RRMS	0	1	none
6	RRMS	0	1	none
7	RRMS	0.2	1	none
8	RRMS	0.1	1	none
9	RRMS	0.5	1	none
10	RRMS	0	1	none
11	RRMS	0	1	none
12	RRMS	0	1	none
13	RRMS	0.1	1	none
14	RRMS	0	1	none
15	RRMS	1	1	none
16	RRMS	12	1	none
17	RRMS	0.1	1	none
18	RRMS	3	2	none
19	RRMS	0	2	none
20	RRMS	2	2	none
21	RRMS	3	2	none
22	RRMS	0	2	none
23	RRMS	0	2	none
24	RRMS	1	2	none
25	RRMS	1	2	none
26	RRMS	0	2	none
27	RRMS	0	2	none
28	RRMS	0.25	2	none
29	RRMS	0	2	none
30	RRMS	0	2	none
31	RRMS	0.2	2	none
32	RRMS	0.25	3	none
33	RRMS	5	3	none
34	RRMS	11	3	none
35	RRMS	5	3	none
36	RRMS	0	3	none
37	RRMS	0.5	3	none
38	RRMS	0	3	none
39	RRMS	0	3	none
40	RRMS	3	3	none
41	RRMS	10	3	none
42	RRMS	0	3	none
43	RRMS	0	3	none
44	RRMS	8	7.5	none
45	RRMS	2	5	none
46	RRMS	0	3.5	none
47	RRMS	20	4	none

Table 1

Supplementary Table 1: Characteristics of individual MS patients and controls.

a, Characteristics of MS patients and controls used in ELISA measurement of TGFA. **b,** Characteristics of MS patients used for multiplex analysis. No additional relevant comorbidities or pharmaceutical treatments were reported in either group.
