# Supplementary material for: TGFα controls checkpoints in CNS resident and infiltrating immune cells to promote resolution of inflammation
Source: Nat Commun. 2025 Jun 19;16:5344. doi: 10.1038/s41467-025-60363-7 (PMC12179293; doi:10.1038/s41467-025-60363-7)
Supplement: Supplementary file 2 — Reporting Summary [file 41467_2025_60363_MOESM2_ESM.pdf]

nature portfolio

Corresponding author(s): Prof. Veit Rothhammer

Last updated by author(s): Apr 30, 2025

Reporting Summary

Nature Portfolio wishes to improve the reproducibility of the work that we publish. This form provides structure for consistency and transparency in reporting. For further information on Nature Portfolio policies, see our Editorial Policies and the Editorial Policy Checklist.

Statistics

For all statistical analyses, confirm that the following items are present in the figure legend, table legend, main text, or Methods section.

n/a	Confirmed	
☐	☒	The exact sample size (n) for each experimental group/condition, given as a discrete number and unit of measurement
☐	☒	A statement on whether measurements were taken from distinct samples or whether the same sample was measured repeatedly
☐	☒	The statistical test(s) used AND whether they are one- or two-sided<br>*Only common tests should be described solely by name; describe more complex techniques in the Methods section.*
☐	☒	A description of all covariates tested
☐	☒	A description of any assumptions or corrections, such as tests of normality and adjustment for multiple comparisons
☐	☒	A full description of the statistical parameters including central tendency (e.g. means) or other basic estimates (e.g. regression coefficient) AND variation (e.g. standard deviation) or associated estimates of uncertainty (e.g. confidence intervals)
☐	☒	For null hypothesis testing, the test statistic (e.g. F, t, r) with confidence intervals, effect sizes, degrees of freedom and P value noted<br>*Give P values as exact values whenever suitable.*
☒	☐	For Bayesian analysis, information on the choice of priors and Markov chain Monte Carlo settings
☒	☐	For hierarchical and complex designs, identification of the appropriate level for tests and full reporting of outcomes
☐	☒	Estimates of effect sizes (e.g. Cohen's d, Pearson's r), indicating how they were calculated

Our web collection on statistics for biologists contains articles on many of the points above.

Software and code

Policy information about availability of computer code

Data collection	Cytek Northern Lights; Cytek SpectroFlo (v3.1.0), Zeiss Zen Blue (v.2012), ClinScan 70/30 (Bruker)
Data analysis	Visualization and statistics were performed using GraphPad Prism (v.9.5.1); FIJI (v1.53); OMIQ, Adobe Illustrator (v.27.6); Adobe Photoshop (v.25.3.1)

For manuscripts utilizing custom algorithms or software that are central to the research but not yet described in published literature, software must be made available to editors and reviewers. We strongly encourage code deposition in a community repository (e.g. GitHub). See the Nature Portfolio guidelines for submitting code & software for further information.

Data

Policy information about availability of data

All manuscripts must include a data availability statement. This statement should provide the following information, where applicable:

- Accession codes, unique identifiers, or web links for publicly available datasets
- A description of any restrictions on data availability
- For clinical datasets or third party data, please ensure that the statement adheres to our policy

Data supporting the findings of this study are available within the supplementary information. Source image data are available from the corresponding author upon request.

Research involving human participants, their data, or biological material

Policy information about studies with human participants or human data. See also policy information about sex, gender (identity/presentation), and sexual orientation and race, ethnicity and racism.

Reporting on sex and gender	Information on sex for the cohorts in Table 1. The sex-distrubution in these cohorts reflects the overall prevalence of Multiple Sclerosis.
Reporting on race, ethnicity, or other socially relevant groupings	Race/ethnicity were not used as constructs for this study. This study was based on biological criteria.
Population characteristics	Information on sex, age, disease duration, disability (EDSS), and treatment can be found in Table 1.
Recruitment	Patients were prespectively recruited in our Neuroimmunology Outpatient Departments for diagnostic procedures including CSF sampling. Patients were only included in this study if the diagnosis of Multiple Sclerosis was confirmed. CSF samples for multiplex and singleplex analyses was obtained from the Biobank at the University Hospital Erlangen.
Ethics oversight	Experiments on human tissue were performed in accordance with the Declaration of Helsinki. The analyses of CSF samples was approved by the standing ethical committee at the ethical committee at the University Hospital Erlangen (20-484_1-Bio and 23-180-Bp).

Note that full information on the approval of the study protocol must also be provided in the manuscript.

Field-specific reporting

Please select the one below that is the best fit for your research. If you are not sure, read the appropriate sections before making your selection.

[x] Life sciences [ ] Behavioural & social sciences [ ] Ecological, evolutionary & environmental sciences

For a reference copy of the document with all sections, see nature.com/documents/nr-reporting-summary-flat.pdf

Life sciences study design

All studies must disclose on these points even when the disclosure is negative.

Sample size	N numbers range from n=3 to n=54, with n= individual mouse/patient, based on previously published work with the same stimulation paradigms and readout (PMID: 36266581; PMID: 29769726). For all cell based experiments, no prior calculations of sample sizes were performed.
Data exclusions	No data was excluded from the analysis.
Replication	For in vitro and in vivo experiments, experiments were repeated at least 3 times. All attempts at replication were successful.
Randomization	Samples and mice were randomly allocated into biological groups.
Blinding	Experimenters were blinded to biological group during EAE scoring. Immunohistochemical analyses were performed blinded. For all other experiments, no blinding was required as it would not affect of the quantitive results.

Reporting for specific materials, systems and methods

We require information from authors about some types of materials, experimental systems and methods used in many studies. Here, indicate whether each material, system or method listed is relevant to your study. If you are not sure if a list item applies to your research, read the appropriate section before selecting a response.

Materials & experimental systems

n/a	Involved in the study	
[ ]	[x]	Antibodies
[ ]	[x]	Eukaryotic cell lines
[x]	[ ]	Palaeontology and archaeology
[ ]	[x]	Animals and other organisms
[x]	[ ]	Clinical data
[x]	[ ]	Dual use research of concern
[x]	[ ]	Plants

Methods

n/a	Involved in the study	
[x]	[ ]	ChIP-seq
[ ]	[x]	Flow cytometry
[x]	[ ]	MRI-based neuroimaging

Antibodies

Antibodies used

Flow Cytometry Antibodies:
BV421-CD11b (Biolegend, #101235; 1:200), BV480-CD11c (BD, #565627, 1:100), BV510-F4/80 (Biolegend,# 123135, 1:100), BV570-Ly6C (Biolegend,# 128029, 1:200), BV605-CD80 (BD, #563052, 1:100), BV650-CD56 (BD, #748098, 1:100), BV650-CD8 (BD, #100741, 1:100), PE-eFlour610-CD140a (Thermo Fisher Scientific, #61140180, 1:100), SuperBright780-MHCII (Thermo Fisher Scientific, #78532080, 1:200), BV711-CD74 (BD, #740748, 1:200), PE-Cy7-Tmem119 (Thermo Fisher Scientific, #25611980, 1:200), eFluor 450-CD3 (Thermo Fisher Scientific, #48003742, 1:200), BV605-CD68 (Biolegend, #137021, 1:200), BV711-CD74 (BD, #740748, 1:200), SB600-CD140B (Thermo Fisher Scientific, #63140282, 1:200), PE-CD105 (Thermo Fisher Scientific, #12-1051-82, 1:100), AF488-A2B5 (Novus Biologicals, #FAB1416G, 1:100), PE-Cy5-CD24 (Biolegend, #101811, 1:200), PE-Cy7-CD31 (Thermo Fisher Scientific, #25031182, 1:200), PerCP-eFlour710-CD86 (Thermo Fisher Scientific, #46086280, 1:100), AF532-CD44 (Thermo Fisher Scientific, #58044182, 1:200), PE-B220 (BD, #561878, 1:100), PE-Cy5.5-CD45 (Thermo Fisher Scientific, #35045180, 1:300), JF646-MBP (Novus Biologicals, #NBP2-22121JF646, 1:100), APC-Cy7-Ly6G (Biolegend, #127623, 1:200), AF700-O4 (R&D, #FAB1326N, 1:200), BUV737-CD154 (BD, #741735, 1:100), AF660-CD19 (Thermo Fisher Scientific, #606019380, 1:100), APC/Fire810-CD4 (Biolegend, #100479, 1:100), PE-eFlour610-iNOS (eBioscience, #61592080, 1:100), BV711-IL17a (Biolegend, #506941, 1:100), PE-Cy5-FoxP3 (Thermo Fisher Scientific, #15-5773-82, 1:200), PE-Cy7-IFNγ (Biolegend, #505826, 1:100), PE PerCP-eFlour710-TNF (eBioscience, #46732180, 1:200), APC-GM-CSF (eBioscience, #17733182, 1:100), PE-CCL2 (BD, #554443, 1:100), PE-IL10 (BD, #554467, 1:200), JF646-TGFα (Novus Biologicals, #NBP2-34683JF646, 1:100), AF700-Ki67 (Thermo Fisher Scientific, #56569882, 1:200).

Immunohistochemistry Antibodies:
mouse anti-TGFα (1:100; Santa Cruz; #sc36-134A), rat anti-GFAP (1:1000; Thermo Fischer Scientific; #2.2B10), rabbit anti-Iba1 (1:1000; Abcam; #ab178846), donkey anti-rabbit IgG AF488 (1:500; Thermo Fisher Scientific; #A21206), donkey anti-mouse IgG AF647 (1:500; Dianova; #715-605-151), goat anti-rat IgG Cy3 (1:500; Thermo Fisher Scientific; #A10522), mouse anti-SMI32 (1:1000; BioLegend; #801701), rabbit anti-Olig2 (1:200; Abcam; #ab109186) or rabbit anti-NeuN (1:500; Abcam; #ab177487), goat anti-rabbit IgG Cy3 (1:500; Thermo Fisher Scientific; #A10520), donkey anti-rabbit IgG AF647 (1:500; Dianova; #711-605-152), rabbit anti-RBPMS (1:300; Merck, ABN1362).

Validation

All commercial antibodies in this study were validated, based on the manufacturers' websites. Antibodies were used for the appropriate animal host and application(s), as per the information provided on those websites:
1. BV421-CD11b (1:200; Biolegend, #101235):
https://www.biolegend.com/en-us/products/brilliant-violet-421-anti-mouse-human-cd11b-antibody-7163?GroupID=BLG10427
• Doni A, et al. 2015. J Exp Med. 212:905.
• Däbritz J, et al. 2016. Sci Rep. 6:20584.
• Chai Y, et al. 2016. PLoS One. 11: 0162853.
• Moderzynski K, et al. 2016. PLoS Negl Trop Dis. .
• Su Y, et al. 2022. J Hematol Oncol. 15:99.
• Hou X, et al. 2020. Cell Reports. 28(1):172-189.e7..
• Liu J, et al. 2019. Immunity. 50:600.
• Ilinykh PA, et al. 2020. Cell Host & Microbe. 27(6):976-991.
• Miller CM, et al. 2020. J Virol. 94:00:00.
• Li Q, et al. 2019. Neuron. 101:207.
• Klemm F, et al. 2020. Cell. 181(7):1643-1660.e17.
• Yan L, et al. 2021. Front Cell Neurosci. 15:750373.

2. BV480-CD11c (1:100; BD, #565627):
https://www.bdbiosciences.com/en-au/products/reagents/flow-cytometry-reagents/research-reagents/single-color-antibodies-ruo/bv480-mouse-anti-human-cd11c.566184
• Knapp W. W. Knapp .. et al., ed. Leucocyte typing IV : white cell differentiation antigens. Oxford New York: Oxford University Press; 1989:1-1182.
• Stacker SA, Springer TA. Leukocyte integrin P150,95 (CD11c/CD18) functions as an adhesion molecule binding to a counter-receptor on stimulated endothelium. J Immunol. 1991; 146(2):648-655. (Clone-specific: ELISA).
• Visser L, Shaw A, Slupsky J, Vos H, Poppema S. Monoclonal antibodies reactive with hairy cell leukemia. Blood. 1989; 74(1):320-325. (Immunogen: Immunocytochemistry (cytospins), Immunohistochemistry, Immunoprecipitation).

3. BV510-F4/80 (1:100; Biolegend, #123135):
https://www.biolegend.com/en-us/products/brilliant-violet-510-anti-mouse-f4-80-antibody-8934
• Schaller E, et al. 2002. Mol. Cell. Biol. 22:8035. (IHC)
• Stevceva L, et al. 2001. BMC Clin Pathol. 1:3. (IHC)
• Kobayashi M, et al.2008. J. Leukoc. Biol. 83:1354.
• Poeckel D, et al. 2009. J. Biol Chem. 284:21077.
• Glass AM, et al. 2013. J. Immunol. 190:4830.
• Koehm S, et al. 2007. J. Allergy Clin. Immunol. 120:570. (IHC)
• Rankin AL, et al. 2010. J. Immunol. 184:1526. (IHC)
• Sasi SP, et al. 2014. J Biol Chem. 289:14178.
• Thakus VS, et al. 2014. Toxicol Lett. 230:322.
• Watson NB, et al. 2015. J Immunol. 194:2796.
• Hirakawa H, et al. 2015. PLoS One. 10:119360.
• Radtke AJ, et al. 2020. Proc Natl Acad Sci U S A. 117:33455-65. (SB)

4. BV570-Ly6C (1:200; Biolegend, #128029):
https://www.biolegend.com/en-us/products/brilliant-violet-570-anti-mouse-ly-6c-antibody-7392
• Harsha Krovi S, et al. 2020. Nat Commun. 4.790277778.
• Sepe JJ, et al. 2022. JACC Basic Transl Sci. 7:915.
• Linnerbauer M, et al. 2022. Front Immunol. 12:800128.
• Li J, et al. 2020. Cancer Discov. .
• Wu X, et al. 2021. Elife. 10:.
• Li J, et al. 2020. Cancer Immunol Res. 0.529166667.
• Haase C, et al. 2022. Nat Methods. 19:1622.
• Stump CT, et al. 2021. Open Biol. 11:210245.
• Ajina R, et al. 2021. Cancer Immunol Res. 9:386.
• Hulsmans M et al. 2017. Cell. 169(3):510-522 .
• Li J, et al. 2018. Immunity. 49:178.
• , et al. 2021. Eur J Immunol. 51:2708.

5. BV605-CD80 (1:100; BD, #563052):
https://www.bdbiosciences.com/en-de/products/reagents/flow-cytometry-reagents/research-reagents/single-color-antibodies-ruo/bv605-hamster-anti-mouse-cd80.563052
• Bluestone JA. New perspectives of CD28-B7-mediated T cell costimulation. Immunity. 1995; 2(6):555-559. (Biology).
• Boussiotis VA, Gribben JG, Freeman GJ, Nadler LM. Blockade of the CD28 co-stimulatory pathway: a means to induce tolerance. Curr Opin Immunol. 1994; 6(5):797-807. (Biology).
• Hathcock KS, Laszlo G, Pucillo C, Linsley P, Hodes RJ. Comparative analysis of B7-1 and B7-2 costimulatory ligands: expression and function. J Exp Med. 1994; 180(2):631-640. (Biology).

6. BV650-CD56 (1:100; BD, #748098):
https://www.bdbiosciences.com/en-de/products/reagents/flow-cytometry-reagents/research-reagents/single-color-antibodies-ruo/bv650-rat-anti-mouse-cd56-ncam-1.748098
• Fujita T, Chen MJ, Li B, et al. Neuronal transgene expression in dominant-negative SNARE mice.. J Neurosci. 2014; 34 (50):16594-604. (Clone-specific: Fluorescence activated cell sorting).
• Li S, Nie EH, Yin Y, et al. GDF10 is a signal for axonal sprouting and functional recovery after stroke.. Nat Neurosci. 2015; 18 (12):1737-45. (Clone-specific: Fluorescence activated cell sorting).
• Rougon G, Deagostini-Bazin H, Hirn M, Goridis C. Tissue- and developmental stage-specific forms of a neural cell surface antigen linked to differences in glycosylation of a common polypeptide.. EMBO J. 1982; 1(10):1239-44. (Biology).

7. BV650-CD8 (1:100; Biolegend, #100741):
https://www.biolegend.com/en-us/products/brilliant-violet-650-anti-mouse-cd8a-antibody-7635
• Schädlich IS, et al. 2022. iScience. 25:104470.
• Flamar AL, et al. 2020. Immunity. 52(4):606-619.e6..
• Wiesner DL, et al. 2020. Cell Host Microbe. 614:27.
• Boyd DF, et al. 2020. Nature. 587:466.
• Kloepper J, et al. 2016. Proc Natl Acad Sci U S A. 113: 4476-4481.
• Schönberger K, et al. 2022. Cell Stem Cell. 29:131.
• Suresh R, et al. 2020. J Immunother Cancer. 8:.
• Arce Vargas F et al. 2018. Cancer cell. 33(4):649-663 .
• Piepke M, et al. 2021. J Neuroinflammation. 18:265.
• Sauter M, et al. 2022. iScience. 25:103677.
• Coleby R, et al. 2021. Clin Exp Rheumatol. :39.
• Abou-Hamad J, et al. 2022. iScience. 25:105524.

8. PE-eFlour610-CD140a (1:100; Thermo Fisher Scientific, #61140180):
https://www.thermofisher.com/antibody/product/CD140a-PDGFRA-Antibody-clone-APA5-Monoclonal/61-1401-80
9. SuperBright780-MHCII (1:200; Thermo Fisher Scientific, #78532080):
https://www.thermofisher.com/antibody/product/MHC-Class-II-I-Ab-Antibody-clone-AF6-120-1-Monoclonal/78-5320-80
10. BV711-CD74 (1:200; BD, #740748):
https://www.bdbiosciences.com/en-de/products/reagents/flow-cytometry-reagents/research-reagents/single-color-antibodies-ruo/bv711-rat-anti-mouse-cd74.740748
• Bertolino P, Rabourdin-Combe C. The MHC class II-associated invariant chain: a molecule with multiple roles in MHC class II biosynthesis and antigen presentation to CD4+ T cells. Crit Rev Immunol. 1996; 16(4):359-379. (Biology).
• Bikoff EK, Huang LY, Episkopou V, van Meerwijk J, Germain RN, Robertson EJ. Defective major histocompatibility complex class II assembly, transport, peptide acquisition, and CD4+ T cell selection in mice lacking invariant chain expression. J Exp Med. 1993; 177 (6):1699-1712. (Biology).
• Bodmer H, Viville S, Benoist C, Mathis D. Diversity of endogenous epitopes bound to MHC class II molecules limited by invariant chain. Science. 1994; 263(5151):1284-1286. (Biology).

11. PE-CD45R/B220 (1:100; BD, #561878):
https://www.bdbiosciences.com/en-de/products/reagents/flow-cytometry-reagents/research-reagents/single-color-antibodies-ruo/pe-rat-anti-mouse-cd45r-b220.561878
• Allman DM, Ferguson SE, Cancro MP. Peripheral B cell maturation. I. Immature peripheral B cells in adults are heat-stable antigenhi and exhibit unique signaling characteristics. J Immunol. 1992; 149(8):2533-2540. (Biology).
• Asensi V, Kimeno K, Kawamura I, Sakumoto M, Nomoto K. Treatment of autoimmune MRL/lpr mice with anti-B220 monoclonal

antibody reduces the level of anti-DNA antibodies and lymphadenopathies. Immunology. 1989; 68(2):204-208. (Clone-specific).
• Ballas ZK, Rasmussen W. Lymphokine-activated killer cells. VII. IL-4 induces an NK1.1+CD8 alpha+beta- TCR-alpha beta B220+ lymphokine-activated killer subset. J Immunol. 1993; 150(1):17-30. (Biology).

12. PE-CD105 (1:100; Thermo Fisher Scientific, #12105182):
https://www.thermofisher.com/antibody/product/CD105-Endoglin-Antibody-clone-MJ7-18-Monoclonal/12-1051-82

13. AF488-A2B5 (1:100; Novus Biologicals, #FAB1416G):
https://www.novusbio.com/products/a2b5-antibody-105_fab1416g
• Á Moreno-Gar, A Bernal-Chi, T Colomer, A Rodríguez-, C Matute, S Mato: Gene Expression Analysis of Astrocyte and Microglia Endocannabinoid Signaling during Autoimmune Demyelination Biomolecules, 2020;10(9):. 2020-01-01 [PMID: 32846891]

14. PE-Cy5-CD24 (1:200; Biolegend, #101811):
https://www.biolegend.com/en-ie/products/pe-anti-mouse-cd24-antibody-343
• Springer T, et al. 1978. Eur. J. Immunol. 8:539. (WB)
• Crowley M, et al. 1989. Cell. Immunol. 118:108. (FA)
• Veillette A, et al. 1989. J. Exp. Med. 170:1671. (FA)
• Pandelakis A Flavell RA 1999 JEM 189:855 (FC, IHC)
• Liu JQ, et al. 2007 J. Immunol. 178:6227. (FC, IF)
• Chappaz S, et al. 2007. Blood doi:10.1182/blood-2007-02-074245. (FC)
• Rucci F, et al. 2010. Proc Natl Acad Sci USA. 107:3024. (FC)
• Teague TK, et al. 2010. Int Immunol. 22:387. (FC)
• Gracz AD, et al. 2010. Am J. Physiol Gastrointest Liver Physiol. 298:590. (FC)
• Chen CY, et al. 2008. Endocrinology. 10:1210. (FC, IHC)
• Qui Q, et al. 2010. J. Immunol. 184:1681. (FC)

15. PE-Cy7-CD31 (1:200; Thermo Fisher Scientific, #25031182):
https://www.thermofisher.com/antibody/product/CD31-PECAM-1-Antibody-clone-390-Monoclonal/25-0311-82

16. PerCP-eFlour710-CD86 (1:100; Thermo Fisher Scientific, #46086280):
https://www.thermofisher.com/antibody/product/CD86-B7-2-Antibody-clone-GL1-Monoclonal/46-0862-80

17. AF532-CD44 (1:100; Thermo Fisher Scientific, #58044182):
https://www.thermofisher.com/antibody/product/CD44-Antibody-clone-IM7-Monoclonal/58-0441-82

18. PE-Cy5.5-CD45 (1:200; Thermo Fisher Scientific, #35045180):
https://www.thermofisher.com/antibody/product/CD45-Antibody-clone-30-F11-Monoclonal/35-0451-80

19. JF646-MBP (1:100; Novus Biologicals, #NBP2-22121JF646):
https://www.novusbio.com/products/mbp-antibody-2h9_nbp2-22121jf646

20. APC-Cy7-Ly6G (1:200; Biolegend, #127623):
https://www.biolegend.com/en-us/products/apc-cyanine7-anti-mouse-ly-6g-antibody-6755
• Fleming TJ, et al. 1993. J. Immunol. 151:2399. (FC)
• Daley JM, et al. 2008. J. Leukocyte Biol. 83:1. (FC)
• Dietlin TA, et al. 2007. J. Leukocyte Biol. 81:1205. (FC)
• Daley J, et al. 2007. J. Leukocyte Biol. doi:10.1189. (Deplete)
• Tadagavadi RK, et al. 2010. J. Immunol. 185:4904.
• Sumagin R, et al. 2010. J. Immunol. 185:7057.
• Guiducci C, et al. 2010. J. Exp Med. 207:2931.
• Fujita M, et al. 2011. Cancer Res. 71:2664.
• Van Leeuwen, et al. 2008. Arterioscler. Thromb. Vasc. Biol. 28:84. (IHC)
• Kowanetz M, et al. 2010. P. Natl. Acad. Sci. USA 107:21248. [supplementary data] (IHC)
• Esbona K, et al. 2016. Breast Cancer Res. 18:35. (IHC)
• Wojtasiak M, et al. 2010. J. Gen. Virol. 91:2158. (FC, Deplete)

21. AF700-O4 (1:200; R&D, #FAB1326N):
https://www.rndsystems.com/products/oligodendrocyte-marker-o4-alexa-fluor-700-conjugated-antibody-o4_fab1326n
• Schachner, M. et al. (1981) Dev. Biol. 83:328.
• Bansal, R. et al. (1989) J. Neurosci. Res. 24:548.
• Bansal, R. and Pfeiffer, S.E. (1989) Proc. Natl. Acad. Sci. USA 86:6181.
• Gard, A. et al. (1995) Dev. Biol. 167:596.
• Reynolds, R. and Hardy, R. (1997) J. Neurosci. Res. 47:455.
• Ono, K. et al. (1997) J. Neurosci. Res. 48:212.
• Pang, Y. et al. (2000) J. Neurosci. Res. 62:510.
• Cai, Z. et al. (2001) Brain Res. 898:126.

22. AF660-CD19 (1:100; Thermo Fisher Scientific, #606019380):
https://www.thermofisher.com/antibody/product/CD19-Antibody-clone-eBio1D3-1D3-Monoclonal/606-0193-80

23. APC/Fire810-CD4 (1:100; Biolegend, #100479):
https://www.biolegend.com/en-us/products/apc-fire-810-anti-mouse-cd4-antibody-19552
• Dialynas DP, et al. 1983. J. Immunol. 131:2445. (Block, IP)
• Dialynas DP, et al. 1983. Immunol. Rev. 74:29. (IP, Deplete)
• Wu L, et al. 1991. J. Exp. Med. 174:1617. (Costim)
• Godfrey DI, et al. 1994. J. Immunol. 152:4783. (Block)
• Gavett SH, et al. 1994. Am. J. Respir. Cell. Mol. Biol. 10:587. (Deplete)
• Schuyler M, et al. 1994. Am. J. Respir. Crit. Care Med. 149:1286. (Deplete)
• Ghobrial RR, et al. 1989. Clin. Immunol. Immunopathol. 52:486. (Deplete)
• Israelski DM, et al. 1989. J. Immunol. 142:954. (Deplete)
• Zheng B, et al. 1996. J. Exp. Med. 184:1083. (IHC)
• Frei K, et al. 1997. J. Exp. Med. 185:2177. (IHC)
• Felix NJ, et al. 2007. Nat. Immunol. 8:388. (Block)
• Radtke AJ, et al. 2020. Proc Natl Acad Sci U S A. 117:33455-65. (SB)

24. PE-eFlour610-iNOS (1:100; Thermo Fisher Scientific, #61592080):
https://www.thermofisher.com/antibody/product/iNOS-Antibody-clone-CXNFT-Monoclonal/61-5920-80

25. BV711-IL17a (1:100; Biolegend, #506941):
https://www.biolegend.com/en-us/products/brilliant-violet-711-anti-mouse-il-17a-antibody-12030
• Kennedy J, et al. 1996. J. Interferon Cytokine Res. 16:611.
• Schubert D, et al. 2004. J. Immunol. 172:4503. (ICFC)
• Infante-Duarte C, et al. 2000. J. Immunol. 165:6107. (ICFC, ELISA Capture)
• Harrington LE, et al. 2005. Nature Immunol. doi:10.1038/ni1254. (ICFC, ELISA Capture)
• Nekrasova T, et al. 2005. J. Immunol. 175:2734. (ELISPOT Capture)
• Yen D, et al. 2006. J. Clin. Invest. 116:1310. (Neut)
• Ehirchiou D, et al. 2007. J. Exp. Med. 204:1519. (ICFC)
• Kang SG, et al. 2007. J. Immunol. 179:3724. (ICFC)
• Smith E, et al. 2008. J. Immunol. 181:1357. (Neut)
• Neufert C, et al. 2007. Eur. J. Immunol. 37:1809.
• Wang C, et al. 2009. Mucosal Immunol 2:173. (ICFC)
• Cui Y, et al. 2009. Invest. Ophth. Vis. Sci. 50:5811. (ICFC)

26. PE-Cy5-FoxP3 (1:100; Thermo Fisher Scientific, #15577382):
https://www.thermofisher.com/antibody/product/FOXP3-Antibody-clone-FJK-16s-Monoclonal/15-5773-82

27. PE-Cy7-IFNγ (1:100; Biolegend, #505826):
https://www.biolegend.com/en-us/products/pe-cyanine7-anti-mouse-ifn-gamma-antibody-5865
• Abrams J, et al. 1992. Immunol. Rev. 127:5. (ELISA, Neut)
• Sander B, et al. 1993. J. Immunol. Meth. 166:201. (ELISA, Neut)
• Abrams J, et al. 1995. Curr. Prot. Immunol. John Wiley and Sons, New York. Unit 6.20. (ELISA, Neut)
• Yang X, et al. 1993. J. Immunoassay 14:129. (ELISA)
• Klinman D, et al. 1994. Curr. Prot. Immunol. John Wiley and Sons, New York. Unit 6.19. (ELISPOT)
• Sander B, et al. 1991. Immunol. Rev. 119:65. (IHC)
• Ferrick D, et al. 1995. Nature 373:255. (FC)
• Ko SY, et al. 2005. J. Immunol. 175:3309. (FC)
• Peterson KE, et al. 2000. J. Virol. 74:5363. (Neut)
• DeKrey GK, et al. 1998. Infect. Immun. 66:827. (Neut)
• Dzhagalov I, et al. 2007. J. Immunol. 178:2113. (ELISA)
• Lawson BR, et al. 2007. J. Immunol. 178:5366. (FC)

28. PE PerCP-eFlour710-TNF (1:100; Thermo Fisher Scientific, #46732180):
https://www.thermofisher.com/antibody/product/TNF-alpha-Antibody-clone-MP6-XT22-Monoclonal/46-7321-80

29. APC-GM-CSF (1:100; Thermo Fisher Scientific, #17733182):
https://www.thermofisher.com/antibody/product/GM-CSF-Antibody-clone-MP1-22E9-Monoclonal/17-7331-82
• Medina-Reyes EI, et al. 2015. Environ Res. 136:424.
• Guillaumond F, et al. 2015. PNAS. 112:2473.
• Sharma SK, et al. 2015. J Immunol. 194:5529.
• Rodero MP, et al. 2014. J. Invest. Dermatol. 7:1991-7.

30. AF700-Ki67 (1:100; BioLegend, #652419):
https://www.biolegend.com/en-us/products/alexa-fluor-700-anti-mouse-ki-67-antibody-10366
• Medina-Reyes EI, et al. 2015. Environ Res. 136:424.
• Guillaumond F, et al. 2015. PNAS. 112:2473.
• Sharma SK, et al. 2015. J Immunol. 194:5529.

• Rodero MP, et al. 2014. J. Invest. Dermatol. 7:1991-7.

31. JF646-TGFα (Novus Biologicals, #NBP2-34683JF646, 1:100):
https://www.novusbio.com/products/tgf-alpha-antibody-mf9_nbp2-34683jf646

Eukaryotic cell lines

Policy information about cell lines and Sex and Gender in Research

Cell line source(s)	HEK293T (Invitrogen, #K1711)<br>HEK293FT (ThermoFisher, #R70007)<br>Human Astrocytes (ScienCell,#1800)<br>N2a cells (CAmerican Type Culture Collection, #CL-131)
Authentication	Cell lines were authenticated prior to receipt by the commercial vendor using the STR-based method
Mycoplasma contamination	Cells tested negative for mycoplasma contamination by the commcercial vendor and upon receipt.
Commonly misidentified lines (See ICLAC register)	No commonly misidentified cell lines were used.

Animals and other research organisms

Policy information about studies involving animals; ARRIVE guidelines recommended for reporting animal research, and Sex and Gender in Research

Laboratory animals	C57BL/6J (The Jackson Laboratory, #000664). Experiments were initiated in 8-12 week old mice.
Wild animals	The study did not involve wild animals.
Reporting on sex	EAE was induced in female mice only due to differences in susceptability and disease severity (PMID: 7517126; PMID: 15081249; PMID: 33190849). No sex-based analysis have been performed. For in vitro experiments, both sex were used.
Field-collected samples	Study did not involve field-collected samples
Ethics oversight	Bavarian State Authoritites (Regierung von Oberbayern, AZ 55.2-2532.Vet_02-19-49; Regierung von Unterfranken, AZ 55.2.2-2532-2-1306, 55.2.2-2532-2-1722, 55.2.2-2532-2-1927).

Note that full information on the approval of the study protocol must also be provided in the manuscript.

Plants

Seed stocks	-
Novel plant genotypes	-
Authentication	-

Flow Cytometry

Plots

Confirm that:

- [x] The axis labels state the marker and fluorochrome used (e.g. CD4-FITC).
- [x] The axis scales are clearly visible. Include numbers along axes only for bottom left plot of group (a 'group' is an analysis of identical markers).
- [x] All plots are contour plots with outliers or pseudocolor plots.
- [x] A numerical value for number of cells or percentage (with statistics) is provided.

Methodology

Sample preparation	Isolation of cells from adult mouse CNS

Mice were perfused with cold 1× PBS and the CNS was isolated and mechanically diced using sterile razors. Their CNS were isolated and mechanically diced using sterile razors. Brains and spinal cords were processed separately, unless otherwise specified, and placed in a 5ml enzyme digestion solution consisting of 35.5 μl papain suspension (Worthington, #LS003126) diluted in enzyme stock solution (ESS), and then equilibrated to 37°C. The ESS was prepared with the following components: 10 ml 10× EBSS (Sigma-Aldrich, #E7510), 2.4 ml 30% D(+)-glucose (Sigma-Aldrich, #G8769), 5.2 ml 1 M NaHCO3 (VWR, #AAJ62495-AP), 200 μl 500 mM EDTA (Thermo Fisher Scientific, #15575020), and 168.2 ml ddH2O, filter-sterilized through a 0.22-μm filter. The samples were then shaken at 80 rpm for 30–40 minutes at 37°C to facilitate enzymatic digestion. Digestion was halted by adding 1 ml of 10× hi ovomucoid inhibitor solution and 20 μl of 0.4% DNase (Worthington, #LS002007) diluted in 10 ml inhibitor stock solution (ISS). The 10× hi ovomucoid inhibitor stock solution contained 300 mg BSA (Sigma-Aldrich, #A8806) and 300 mg ovomucoid trypsin inhibitor (Worthington, #LS003086) diluted in 10 ml 1× PBS and filter-sterilized using a 0.22-μm filter. The ISS contained 50 ml 10× EBSS (Sigma-Aldrich, #E7510), 6 ml 30% D(+)-glucose (Sigma-Aldrich, #G8769), and 13 ml 1 M NaHCO3 (VWR, #AAJ62495-AP) diluted in 170.4 ml ddH2O and filter-sterilized through a 0.22-μm filter. The homogenized tissue was then mechanically dissociated using a 5-ml serological pipette and triturated through a 70-μm cell strainer (Fisher Scientific, #22363548) into a fresh 50-ml conical tube. Following this, the tissue was centrifuged at 600g for 5 minutes and resuspended in 10 ml of 30% Percoll solution (9 ml Percoll from GE Healthcare Biosciences, #17-5445-01, 3 ml 10× PBS, and 18 ml ddH2O) for purification. The Percoll suspension was centrifuged at 600g for 25 minutes with no brakes. After discarding the myelin top layer and supernatant, the remaining mononuclear cell pellet was washed once with 1× PBS, centrifuged at 500g for 5 minutes, and prepared for further applications.

Isolation of splenic cells
The spleens were mechanically dissected, and the tissue was further dissociated by triturating it through a 100-μm cell strainer (Fisher Scientific, 10282631). To remove red blood cells, an ACK lysing buffer (Life Technology, A10492-01) was applied for 5 minutes. Subsequently, the cells were washed with a solution containing 0.5% BSA and 2 mM EDTA at pH 8.0 in 1× PBS. After this process, the cells were prepared for use in further applications

Instrument	Cytek Northern Lights (Cytek)
Software	Cytek SpectroFlo (v3.1.0)
Cell population abundance	For cell population abundances see the respective figures. Population abundances are depicted as percent of live cells.
Gating strategy	Following FSC/SSC discrimination and doublet exclusion (SSC-H/SSC-A), hematopoetic/myeloid cells were differentiated from non hematopoetic/myeloid cells by CD45/CD11b. Subsets were then further gated based on the expression of specific surface markers.

☒ Tick this box to confirm that a figure exemplifying the gating strategy is provided in the Supplementary Information.
